# Effect of Nano-SnS and Nano-MoS_2_ on the Corrosion Protection Performance of the Polyvinylbutyral and Zinc-Rich Polyvinylbutyral Coatings

**DOI:** 10.3390/nano9070956

**Published:** 2019-06-30

**Authors:** Zuopeng Qu, Lei Wang, Hongyu Tang, Huaiyu Ye, Meicheng Li

**Affiliations:** 1 National Engineering Laboratory for Biomass Power Generation Equipment, School of Renewable Energy, North China Electric Power University, Beijing 102206, China; 2 Delft Institute of Microsystems and Nanoelectronics, Delft University of Technology, 2628 CD Delft, The Netherlands; 3 Changzhou Institute of Technology Research for Solid State Lighting, Changzhou 213161, China; 4 State Key Laboratory of Alternate Electrical Power System with Renewable Energy Sources, School of Renewable Energy School, North China Electric Power University, Beijing 102206, China

**Keywords:** anti-corrosion, tin sulfide (SnS), molybdenum disulfide (MoS_2_), electrochemical test, composite coating

## Abstract

In this paper, four composite coatings of nano-SnS/polyvinylbutyral (PVB), nano-MoS_2_/PVB, nano-SnS-Zn/PVB, and nano-MoS_2_-Zn/PVB were prepared, and their anti-corrosion mechanism was analyzed by experimental and theoretical calculations. The results of the electrochemical experiments show that the effect of nano-MoS_2_ on the corrosion protection performance of PVB coating is better than that of nano-SnS in 3% NaCl solution, and that the addition of Zn further enhances this effect, which is consistent with the results of weight loss measurements. Furthermore, the observation of the corrosion matrix by the field emission scanning electron microscope (FESEM) further confirmed the above conclusion. At last, the molecular dynamics (MD) simulation were carried out to investigate the anti-corrosion mechanism of the nanofillers/PVB composites for the copper surface. The results show that both nano-SnS and nano-MoS_2_ are adsorbed strongly on the copper surface, and the binding energy of nano-MoS_2_ is larger than that of nano-SnS.

## 1. Introduction

Copper has good thermal and electrical properties, and is a commonly used material in marine engineering. It is often used in many parts such as hulls, pipes, electronic devices, etc. [1,2,3]. However, the corrosive nature of seawater and gas above the ocean severely limits the service life of copper [4,5] and brings additional costs. Therefore, it is very meaningful to find a good way to delay the corrosion of copper. The protection of copper can be carried out in various ways such as inhibitor [6,7], film [8], coating [9,10]. The method of applying a composite coating to a metal substrate is convenient in application and low in cost, and is suitable for mass production in the industry. Thus many researchers have added various nanoparticles as fillers in polyvinylbutyral (PVB) as a physical barrier to enhance the barrier effect of the coating on water, oxygen, and other corrosive media [10].

Conventional graphene materials have been used as fillers in anti-corrosion coatings due to their high surface-to-volum ratio and excellent physical properties. However, they have a ‘corrosion-promoting activity’ when the coating is broken because of good electrical conductivity [9,11], which promotes the corrosion of the metal substrate. As a semiconductor material, tin sulfide (SnS) has good chemical stability and will not cause harm to human health and environment [12]. It is widely used in optoelectronics and sensors [13,14,15,16]. However, there are currently few reports on anti-corrosion applications for SnS. SnS has a very large resistivity and therefore may exhibit better corrosion resistance compared to graphene. MoS_2_ is a transition metal disulfide with a variety of excellent physical properties. It has a layered structure and can be used to construct a variety of one-, two-, and three-dimensional materials [17,18,19,20,21,22,23], which has broad application prospects. In addition, MoS_2_ has good hydrophobicity [24,25], which has been used by researchers to make composite coatings or to modify existing graphene products [17,26]. Therefore, we consider adding nano-MoS_2_ as a filler to the coating to verify its protection against metallic copper. Moreover, nano-Zn, as an active metal, can be used as a cathodic protection in the coating to improve the protective ability of the coating. At present, many researchers have used Zn or zinc ions as additives to modify coatings [27,28] to enhance the protection of metals.

Our current work aims to study and compare the corrosion protection performance of nano-SnS and nano-MoS_2_ on polyvinylbutyral (PVB) coatings and zinc-rich PVB coatings. Weight loss, potentiodynamic polarization, and electrochemical impedance spectroscopy (EIS) were used to evaluate the anti-corrosion performance of nano-SnS/PVB, nano-MoS_2_/PVB, nano-SnS-Zn/PVB and nano-MoS_2_-Zn/PVB coatings at first. In addition, field emission scanning electron microscope (FESEM) was used to characterize the morphology of the copper after corrosion to verify the test results. Furthermore, molecular dynamics (MD) simulations were used to study the adsorption properties of nano-SnS/PVB and nano-MoS_2_/PVB on copper surfaces.

## 2. Experimental

### 2.1. Material and Sample Preparation

Some of the key materials used in this experiment are shown in Table 1, in which the average particle size of the nanoparticles is 50 nm.

**Figure 1:**
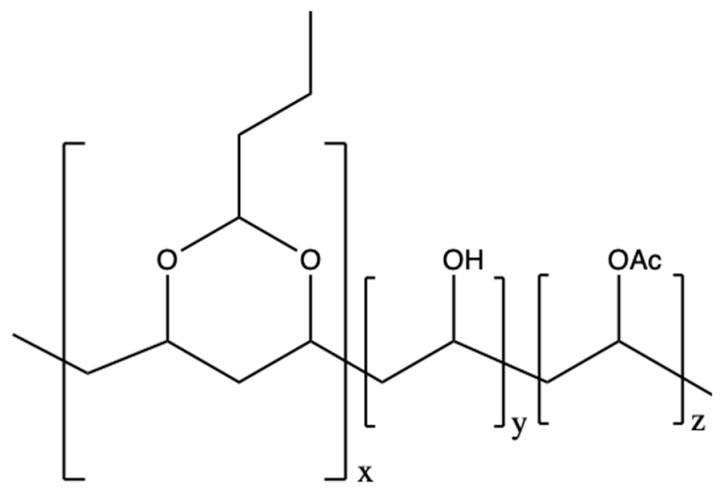
Molecular structure of PVB used in this work.

Nano-SnS (0.1 g) was sonicated in 10 mL of methanol for more than 5 h to form a suspension. Then, 1.0 g of PVB powder was added to the suspension, and the mixture was thoroughly stirred for more than 36 h on a magnetic stirrer to prepare a uniform paint, and the resultant material was allowed to stand for use. The molecular structure of PVB is shown in Figure 1. Nano-MoS_2_/PVB, nano-SnS-Zn/PVB, and nano-MoS_2_-Zn/PVB coatings were prepared in the same manner, wherein the amount of Zn added was 0.05 g. The copper piece with a thickness of 0.05 mm was cut to a size of 1.0 cm × 1.0 cm, and it was carefully polished with 400, 800, and 2,000 mesh emerald paper. After the sanding was completed, the copper sheets were ultrasonically cleaned with ultrapure water for more than 5 min, then degreased with acetone and air dried naturally in a fume hood. The copper sheet was dipped into the prepared paint for 40 s and then taken out at a rate of about 0.5 mm s^−1^. The samples were air dried naturally in a fume hood for 24 h. The thickness of the prepared coatings was essentially the same and they were controlled at 19.3 ± 1.2 μm, which was measured with a portable coating thickness gauge (EC-770, YOWEXA, Shenzhen, China). Some of the copper sheets were sealed with 705 silicone rubber on the back, leaving 1.0 cm^2^ of working surface for electrochemical experiments, while the rest retained double working faces for weight loss measurements. A NaCl solution having a concentration of 3.0% was prepared for electrochemical experiments and weight loss measurements. All experiments were performed at room temperature (about 293 K).

### 2.2. Weight Loss Measurements

Weight loss measurements were carried out in glass dishes at 293 K. Samples, without and with different coatings, prepared in triplicate were each immersed in a 3.0% NaCl solution. The samples were continuously immersed for 35 days, then soaked in methanol to dissolve the surface coating and thoroughly rinsed in 0.1 M HCl, water, and acetone in order to remove corrosion products and other impurities. The copper sheets were weighed with an analytical balance after drying. The average corrosion rate was finally calculated by the immersion time and the weight loss of each sample.

### 2.3. Electrochemical Experiments

The electrochemical experiments in this paper are performed using a conventional three-electrode system. The copper piece to be tested was the working electrode, the saturated calomel electrode (SCE) was used as the reference electrode, and the platinum mesh electrode (2.0 cm × 2.0 cm) was used as the counter electrode. The CHI660C electrochemical workstation was used for the electrochemical measurements. Prior to each test, the working electrode was immersed in an etching medium for about 30 min at an open circuit potential (OCP) until the OCP value reached an almost constant state. EIS measurements were made under open circuit potential with a sweep frequency range of 10 mHz–100 kHz and an amplitude of 5 mV AC sinusoidal disturbance. The EIS data were analyzed and fitted by Zsimpwin 3.60 software. Furthermore, a polarization experiment was performed at a potential range of ±250 mV relative to the OCP at a scan rate of 2 mV s^−1^. Each sample was tested more than 4 times to ensure the reproducibility of the experiments.

### 2.4. Scanning Electron Microscope (SEM) Observations

Scanning electron microscopy (SEM, JSM6490LV, JEOL, Tokyo, Japan) was used to observe and analyze the copper matrix without and with different coatings at 293 K. Prior to observation, all samples were immersed in a 3.0% NaCl solution at 293 K for 10 days, and then the surface coated samples were immersed in anhydrous methanol to remove the surface coating.

### 2.5. Theoretical Study

In order to further discuss the interaction between the PVB/nanosheets composites and copper surface, molecular dynamics (MD) simulation were carried out to model the adsorption structure of SnS-PVB and MoS_2_-PVB system on copper (111) surface. MD simulations were performed using commercially available software, Material Studio 8.0, purchased from Accelrys Inc (San Diego, CA, USA). The structure of PVB chain and molecules of SnS or MoS_2_ nanosheet were generated and optimized through the Forcite module. The PVB model was prepared with the dimensions of 28 × 30 × 126 Å, with an initial density of 1.05 g cc^−1^, which agrees well with experimental data of 1.07 g cc^−1^ [29]. The energy minimized structures of PVB (2 chains) and nanosheets were used for the construction of different amorphous cells. The optimization procedure follows convergence criteria: 2.0 × 10^−5^ kcal mol^−1^ for energy, 0.001 kcal mol^−1^ Å^−1^ for force, and 1.0 × 10^−5^ for displacement. After the geometry optimization, molecular dynamic calculation with constant number of particles, volume and temperature (NVT) and Universal force field (UFF) [30] was performed at time step of 1.0 fs up to total 3.0 ns, among which Andersen algorithm themostat with 1.0 Collision ratio was used to maintain the temperature of the system at around 298 K. The interaction energy $E_{\mathrm{Interaction}}$, post to equilibration is then calculated using Equation (1):

(1) $$E_{\mathrm{Interaction}}{\;=E}_{\mathrm{Total}}\;-{(E}_{\mathrm{Cu}\;}{+E}_{\mathrm{composite}}),$$

where $E_{\mathrm{Total}}$ is the combined energy of the Cu surface and the PVB-nanosheets system, $E_{\mathrm{Cu}\;}$ is the energy of the solo Cu surface, and $E_{\mathrm{composite}}$ is the energy of the PVB-nanosheets system taken independently. Based on Equation (1), it can be stated that the more negative the value, the better is the adhesion the composites coating applied on the surface.

## 3. Results and Discussion

### 3.1. Weight Loss Measurements

The corrosion resistance of various coatings to copper was investigated by weight loss measurements after immersion in a 3.0% NaCl solution at 293 K for 35 days. The corrosion rates (*ω*, mg m^−2^ h^−1^) and protective efficiency ($\eta_{w}$) of these coatings were calculated as follows, and the results are shown in Table 2,

(2) $$\omega =\frac{m_{0}-m}{A\tau },$$

(3) $$\eta_{w}\%=\frac{\omega_{0}-\omega }{\omega_{0}}\cdot 100,$$

where *A* is the total surface area of the sample; $m_{0}$, and m are the weights of the sample before and after immersion in the corrosive solution, respectively; *τ* is the soaking time; and $\omega_{0}$ and *ω* are the corrosion rates of the copper samples containing and containing the coating, respectively.

As shown in Table 2, the corrosion rate of the coated copper sheet sample was smaller than that of the uncoated copper sheet sample. Compared to pure PVB coatings, copper samples with nanofiller coatings have a lower corrosion rate and higher protection efficiency. Furthermore, the addition of Zn can improve the protection efficiency of the coating by more than 10%. It is worth noting that for the nano-MoS_2_-Zn/PVB coating, the $\eta_{w}$ value is as high as 76%, which is 46% higher than the pure PVB coating.

### 3.2. Polarization Curve

**Figure 2:**
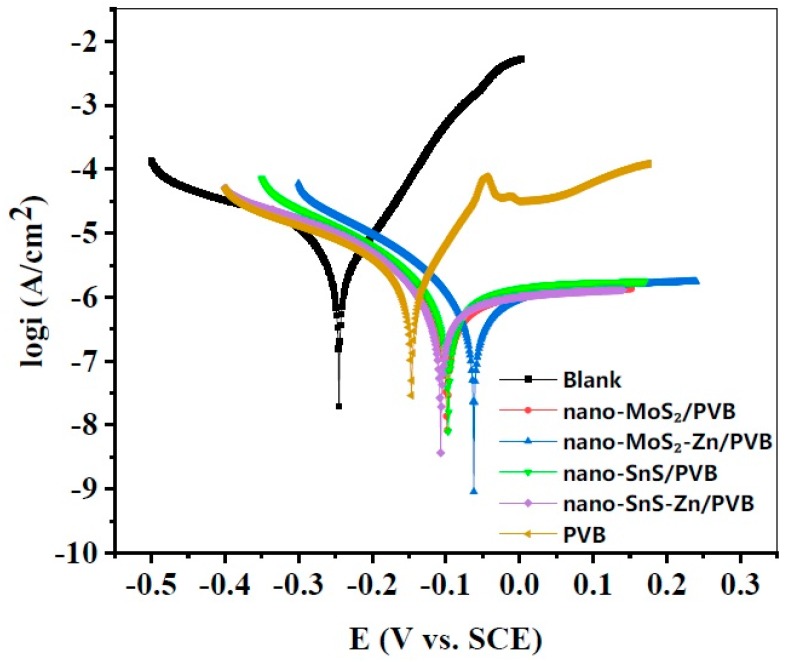
Tafel curve recorded for copper samples without and with different coatings in 3.0% NaCl solution at 293 K.

The Tafel polarization curves of copper electrodes coated with different coatings measured in 3% NaCl solution are shown in Figure 2. We obtained the main electrochemical parameters by extrapolation, including corrosion potential (*E_corr_*), corrosion current density (*i_corr_*), anode and cathode Tafel slope (*β_a_, β_c_*), and protection efficiency ($\eta_{P}$). Their values were listed in Table 3. The value of the protection efficiency *η* is calculated as follows,

(4) $$\eta_{P}(\%)=\frac{i_{\mathrm{corr},o}-i_{\mathrm{corr},k}}{i_{\mathrm{corr},0}}\cdot 100,$$

where $i_{\mathrm{corr},o}$ (A cm^−2^) is the corrosion current density of the uncoated copper sample, $i_{\mathrm{corr},k}$ (A cm^−2^) is the corrosion current density of the sample containing the different composite coating.

Figure 2 shows that the current densities of the samples with coatings are significantly lower than that of the uncoated copper. More importantly, the coating with the nanofiller exhibited a significantly lower current density than the pure PVB coating. This indicates that after the surface of the copper sheet is coated, its corrosion strength and corrosion rate, in 3% NaCl becomes lower.

According to Table 3, the current density (*i_corr_*) of MoS_2_ is lower than that of SnS, and the *i_corr_* higher compared with the corresponding Zn-containing composite coatings of SnS and MoS_2_. This indicates that the corrosion protection ability of MoS_2_ is better than that of SnS, and the addition of Zn has a certain improvement effect on the protective ability of the coating. The $\eta_{P}$ value of the sample with the composite coating increased by more than 40% compared to the sample with a pure PVB coating, and the efficiency value of the nano-MoS_2_-Zn/PVB coating was the largest, reaching 72.9%. The experimental results show that the composite coating has a significant protective effect on the metal matrix, which is in line with the result of weight loss measurements.

### 3.3. Electrochemical Impedance Spectroscopy (EIS)

**Figure 3:**
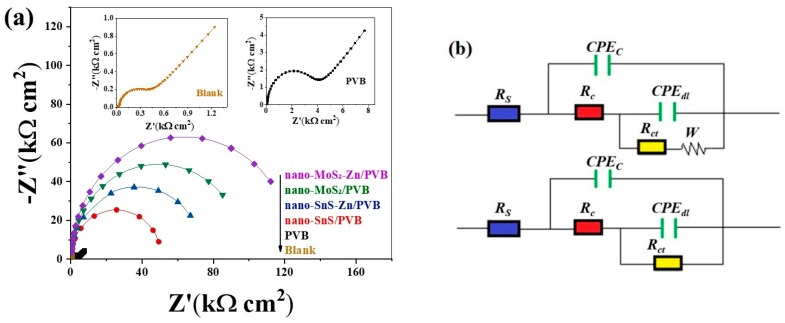
(**a**) Nyquist plots for copper samples in 3.0% NaCl solution without and with different coatings at 293 K and (**b**) equivalent circuit diagrams for fitting Electrochemical Impedance Spectroscopy (EIS) data.

In order to study the corrosion mechanism of the metal and the improvement of the corrosion resistance of the coating, we measured the electrochemical impedance spectroscopy of copper with pure PVB coatings and different nanofiller composite coatings. The experiments were carried out in 3.0% NaCl solution. As shown in Figure 3a, the Nyquist plot of the PVB coated and uncoated copper sheet show an incomplete semicircle in the high frequency region and an approximate straight line in the subsequent low frequency range. In general, the high frequency region semicircle is related to the charge transfer resistance (*R_ct_*) and the double layer capacitance (*C_dl_*). The low frequency impedance is the Warburg impedance (*W*), which means the diffusion of dissolved oxygen or soluble cuprous chloride complexes during the corrosion process.

Figure 3a shows that after the addition of the nanofiller, the shape of the curve appears to be approximately semicircular, and the Warburg impedance in the low frequency region disappears. This indicates that the addition of nanofiller inhibits the diffusion of dissolved oxygen and cuprous chloride complexes. At this time, the corrosion of copper depends on the charge transfer process. The order of the diameter of the semicircle obtained by different coating samples is: nano-MoS_2_-Zn/PVB > nano-MoS_2_/PVB > nano-SnS-Zn/PVB > nano-SnS/PVB, and the diameter of the semicircle of the sample added with Zn is obviously increased. According to this result, it can be judged that the nano-MoS_2_ filler is better than the nano-SnS for the corrosion protection performance of the PVB coatings and the addition of Zn particles further improves the corrosion resistance of the composite coatings.

In order to quantitatively compare the corrosion inhibition properties of different coatings, we further fit the EIS data using the equivalent circuit diagram shown in the inset of Figure 3b, and the resulting electrochemical parameters are listed in Table 4, where *R_s_* is the solution resistance, *R_c_* is the resistance of the coating on the copper working surface, *R_ct_* is the charge transfer resistance, and *W* is the Warburg impedance. *CPE_c_* and *CPE_dl_* are constant phase angle elements representing the coating capacitance (*C_c_*) and the double layer capacitance (*C_dl_*), respectively. The impedance of these circuits can be expressed as follows [31],

(5) $$Z_{W}{=R}_{S}+\frac{1}{{\mathrm{jwCPE}}_{c}+\frac{1}{R_{c}}+\frac{1}{{\mathrm{jwCPE}}_{\mathrm{dl}}+\frac{1}{R_{\mathrm{ct}}+w}}}$$

(6) $${Z\;=R}_{S}+\frac{1}{{\mathrm{jwCPE}}_{c}+\frac{1}{R_{c}}+\frac{1}{{\mathrm{jwCPE}}_{\mathrm{dl}}+\frac{1}{R_{\mathrm{ct}}}}}$$

The impedance of the *CPE* is defined as follows [31],

(7) $$Z_{\mathrm{CPE}}\;=\frac{w^{-n}}{Y{\left(\frac{\cos n\pi }{2}+j\frac{\sin n\pi }{2}\right)}^{n}}$$

where *Y* is the modulus of the *CPE*, *w* is the angular frequency, *j* is the imaginary number, and *n* is the deviation parameter. The *η* values of the copper electrode coatings in these 3% NaCl solutions are calculated as follows,

(8) $$\eta_{E}(\%)=\frac{R_{\mathrm{ct}}-R_{\mathrm{ct},0}}{R_{\mathrm{ct}}}\cdot 100$$

where $R_{\mathrm{ct}}$ and $R_{\mathrm{ct},0}$ are the charge transfer resistances of copper samples with and without various coatings in 3% NaCl solution, respectively.

As can be seen from Table 4, the *R_c_* and *R_ct_* values of the nano-MoS_2_/PVB coated samples were larger than those of nano-SnS/PVB, and these values improved in the samples to which Zn was added. This again demonstrates that nano-MoS_2_ enhances the corrosion resistance of the coating better than nano-SnS, and the addition of Zn further enhances the corrosion resistance of the coating. In addition, all copper samples with coatings incorporating nanofillers had $\eta_{E}$ values above 99%, which is a significant increase compared to samples with pure PVB coatings. These demonstrate that the use of nanoparticles as a filler can effectively enhance the corrosion resistance of polymer coatings. The trend of these values is basically consistent with the weight loss measurements results. Several typical corrosion resistant materials and their values of $\eta_{E}$ obtained in a NaCl solution are listed in Table 5. The higher $\eta_{E}$ values further highlight the superior corrosion resistance of the composite coatings in this study compared to these materials [6,32,33].

### 3.4. SEM Analyses

**Figure 4:**
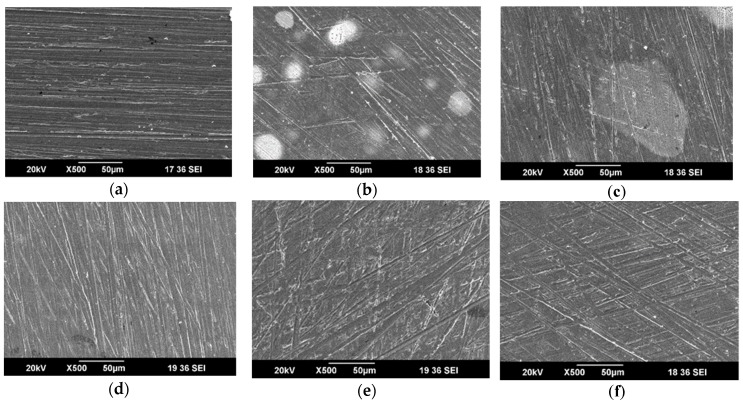
SEM images of (**a**) freshly polished copper specimen and the specimens immersed in 3% NaCl solution with (**b**) PVB, (**c**) nano-SnS/PVB, (**d**) nano-SnS-Zn/PVB, (**e**) nano-MoS_2_/PVB, and (**f**) nano-MoS_2_-Zn/PVB coating for10 days at 293 K.

At 293 K, SEM high-resolution photographs of copper samples without and with different coatings immersed in 3% NaCl solution for 10 days are shown in Figure 4. After the copper with pure PVB coating (Figure 4b) was immersed in 3% NaCl solution, the substrate experienced severe corrosion with many obvious large-area corrosion marks. Although the nano-SnS/PVB sample (Figure 4c) also showed local corrosion, the degree of corrosion was lower than that of the pure PVB coating sample, and large-area local corrosion disappeared after the addition of Zn (Figure 4d). In addition, the nano-MoS_2_/PVB sample (Figure 4e) exhibited slight signs of corrosion, while the substrate surface of the nano-MoS_2_-Zn/PVB sample (Figure 4f) was well protected.

The observation of these high-definition pictures proves that MoS_2_ can improve the anti-corrosion performance of PVB more than SnS, and the addition of Zn further enhances this performance. The SEM analysis further validated the results of electrochemical experiments and weight loss measurements.

### 3.5. Corrosion Mechanism Analysis

The corrosion process of Cu in NaCl solution has been described in numerous reports [34,35,36,37]. As we all know, in the corrosion process of copper with NaCl solution as the medium, the cathodic reaction is represented by the reduction of oxygen [1,38],

(9) $$O_{2}{+4e+2H}_{2}O\to {4\mathrm{OH}}^{-}$$

The anode undergoes the following series of complex reactions [39],

(10) $$\mathrm{Cu}\to {\mathrm{Cu}}^{+}+e,$$

(11) $${\mathrm{Cu}}^{+}{+\mathrm{Cl}}^{-}\to \mathrm{CuCl},$$

(12) $${\mathrm{CuCl}+\mathrm{Cl}}^{-}\to {\mathrm{CuCl}}_{2}^{-},$$

(13) $${\mathrm{CuCl}}_{2}^{-}\to {\mathrm{Cu}}^{2+}{+2\mathrm{Cl}}^{-}+e$$

The insulation of the composite coating inhibits the transfer of current, hinders the formation of a closed loop between the substrate and the etching solution, and reduces the corrosion rate. Besides, the nanoparticle filler acts as a physical barrier in the polymer, preventing the penetration of O_2_ and H_2_O into the metal, and the diffusion of ${\mathrm{CuCl}}_{2}^{-}$ into the 3% NaCl solution. Moreover, for the Zn-added coating, since Zn has a lower electronegativity than Cu, Zn is first corroded in the system, which delays the oxidation process of the anode Cu as shown in Equation (10). This further explains the test results of the EIS. The analysis of corrosion and protection mechanisms is consistent with Han et al. [9,40].

### 3.6. Molecular Dynamics Simulation

**Figure 5:**
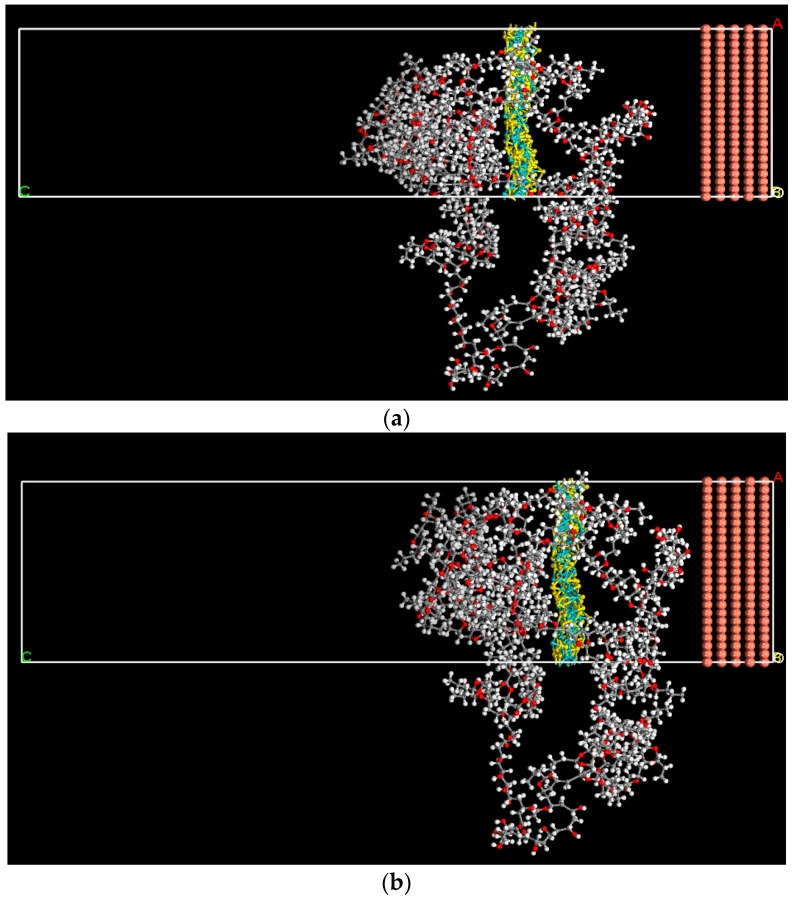
Illustration of (**a**) the MoS_2_-PVB system consisting of copper (111) crystal as the substrate (shown in brown spheres) and PVB containing 10% MoS_2_ at time 0 ps. (**b**) Final MoS_2_-PVB system post-MD run of 500 ps.

**Figure 6:**
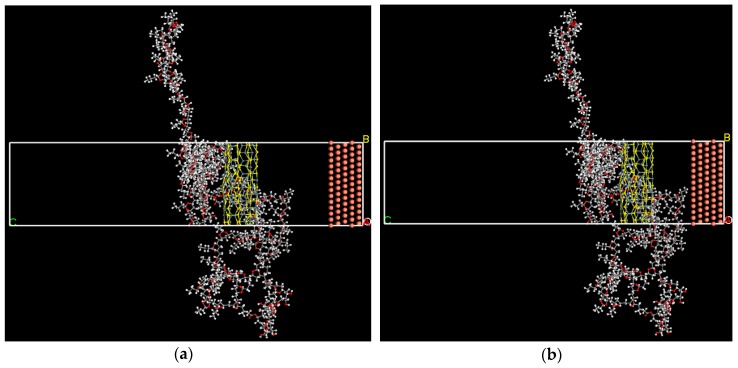
Illustration of (**a**) the SnS-PVB system consisting of copper (111) crystal as the substrate (shown in brown spheres) and PVB containing 10% SnS at time 0 ps. (**b**) Final SnS-PVB system post-MD run of 500 ps.

As shown in Figure 5 and Figure 6, after sufficient relaxation and of equilibrium the PVB-nanosheets system, two types of composites slowly approached to Cu surface, indicating that the combined energy of the Cu surface and the PVB-nanosheets system is large. The interaction energy $E_{\mathrm{Interaction}}$ of pure PVB, SnS/PVB, MoS_2_/PVB with Cu surface are listed in Table 6. All the energies obtained have been found to be negative in sign, which means that all the formulated coatings show sufficient binding to the surface. The $E_{\mathrm{Interaction}}$ of MoS_2_/PVB composite (−1,838.253 kcal mol^−1^) is larger than that of SnS/PVB composites (−1,074.433 kcal mol^−1^) and PVB coatings (−852.33 kcal mol^−1^), indicating that the anti-corrosion behavior of MoS_2_/PVB composite coatings is better than that of SnS/PVB and pure PVB. The MD simulation results are in good agreement with the results obtained from potentiodynamic polarization measurements and EIS, which is further confirm that the excellent anti-corrosion performance of MoS_2_/PVB is attributed to its high interfacial binding energy. In addition, the $E_{\mathrm{Interaction}}$ is larger than that of previous work, such as −500 kcal mol^−1^ of Polyvinyl acetate (PVAc)-Perfluorooctane (PFO) systems and −27.3 kcal mol^−1^ graphene-based polymer coatings [41,42], which reveals the MoS_2_/PVB and SnS/PVB composites coatings exhibit excellent corrosion protection performance than many nanosheets-polymer composites.

## 4. Conclusions

The anti-corrosion performance of nano-SnS/PVB, nano-MoS_2_/PVB, nano-SnS-Zn/PVB, and nano-MoS_2_-Zn/PVB was studied by experiments and theoretical calculations. All four coatings have good corrosion protection performance and their protective efficiencies calculated from the weight loss and polarization curves are consistent. Moreover, the Nyquist plot and fit to the EIS data indicate that nano-MoS_2_/PVB has better anti-corrosion performance than nano-SnS/PVB. The addition of Zn further enhances this performance. These can be further confirmed by FESEM observation. Compared to graphene-based films or other composite coatings with nanomaterials as fillers, the composite coatings prepared herein have higher $\eta_{P}$ and *R_ct_* values [43,44], meaning that the coatings have better corrosion resistance. At last, the results of the molecular dynamics (MD) simulation show that both nano-SnS and nano-MoS_2_ are adsorbed strongly on the copper surface, and the binding energy of nano-MoS_2_ is larger than that of nano-SnS. Furthermore, compared to some other studies, these composite coatings have a larger interaction energy $E_{\mathrm{Interaction}}$ with the copper surface. There are still many aspects of this research that need to be explored and improved in future work, such as sample preparation methods and coating modification.

## Figures and Tables

**Table 1:** Materials used for preparing composite coatings.

Material	Manufacturer	Label
Polyvinylbutyral	MACKLIN	P815775
Tin sulfide	6Carbon Tech. Shenzhen	SC-CRYSTAL-SNS
Molybdenum disulfide	HANLANE	MoS_2_-50
Zinc	HANLANE	Zn-50

**Table 2:** Corrosion rate and protection efficiency of copper sheets without and with different coatings in 3.0% NaCl solution at 293 K.

Coating	*ω* (mg m^−2^ h^−1^)	*η_w_* (%)
Blank	59.52	-
PVB	41.67	30
nano-SnS	23.81	60
nano-SnS-Zn/PVB	15.48	74
nano-MoS_2_/PVB	20.24	66
nano-MoS_2_-Zn/PVB	14.29	76

**Table 3:** Electrochemical parameters for copper sample without and with different coatings in 3.0% NaCl solution at 293 K.

Coating	*E_corr_* (mV per SCE)	*i_corr_* (μA cm^−^^2^)	*β_c_* (mV dec^−^^1^)	*β_a_* (mV dec^−^^1^)	Crate (mpy)	$\eta_{P}\;(\%)$
Blank	−245	2.185	60	55	1.00	-
PVB	−147	1.839	179	75	0.85	15.8
nano-SnS/PVB	−97	0.921	105	280	0.42	57.9
nano-SnS-Zn/PVB	−107	0.757	99	317	0.35	65.4
nano-MoS_2_/PVB	−98	0.710	102	297	0.33	67.5
nano-MoS_2_-Zn/PVB	−62	0.591	84	190	0.27	72.9

**Table 4:** Electrochemical parameters of EIS in copper samples without and with different coatings in 3.0% NaCl solution at 293 K.

Coating	*R_s_* (Ω cm^2^)	*R_c_* (kΩ cm^2^)	*R_ct_* (kΩ cm^2^)	*C_c_* (μF cm^−2^)	*C_dl_* (μF cm^−2^)	*W*	$\eta_{E}\;(\%)$
Blank	7.94	0.04	0.32	13.08	128.30	0.002902	-
PVB	19.50	0.07	3.50	1.84	18.17	0.000620	90.86
nano-SnS/PVB	18.14	0.11	50.84	2.69	54.21	-	99.37
nano-SnS-Zn/PVB	22.54	0.30	74.36	2.49	69.49	-	99.57
nano-MoS_2_/PVB	20.07	0.26	97.84	3.07	60.39	-	99.67
nano-MoS_2_-Zn/PVB	25.09	0.30	126.20	1.92	43.34	-	99.75

**Table 5:** Anti-corrosion materials and their protection efficiency ($\eta_{E}$) by EIS test in 3.0–3.5% NaCl solution.

Classification	Samples	$\eta_{E}\;(\%)$
Silicon carbide composite	POA–SiC/EP	87.54
Metal Organic Framework	ATT/ZIF-8	97.3
Organic inhibitor	polyaspartic acid	86.8

**Table 6:** The simulated surface energy for different systems at 298 K.

System	Interaction Energy (kcal mol^−^^1^)
Cu+PVB	−852.33
Cu+MoS_2_/PVB	−1838.253
Cu+SnS/PVB	−1074.433
